# Opticin Ameliorates Hypoxia-Induced Retinal Angiogenesis by Suppression of Integrin α2-I Domain–Collagen Complex Formation and RhoA/ROCK1 Signaling

**DOI:** 10.1167/iovs.63.1.13

**Published:** 2022-01-10

**Authors:** Xiaoxue Liu, Yue Xing, Xin Liu, Lingyan Zeng, Jin Ma

**Affiliations:** 1 State Key Laboratory of Ophthalmology, Zhongshan Ophthalmic Center, Sun Yat-sen University, Guangzhou, China

**Keywords:** opticin, collagen, integrin α2-I domain, RhoA/ROCK1, Mg^2+^

## Abstract

**Purpose:**

It was previously demonstrated that opticin (*OPTC*) inhibits the collagen-induced promotion of bioactivities of human retinal vascular endothelial cells (hRVECs). The present in vivo study aimed to further investigate the regulatory role of opticin in vitreous collagen-mediated retinal neovascularization and to elucidate its regulatory mechanisms with regard to integrin α2-I domain–GXXGER complex formation and RhoA/ROCK1 signal change. The regulatory role of Mg^2+^ on integrin α2-I domain–GXXGER complex formation in the above process was also investigated.

**Methods:**

The zebrafish model of hypoxia-induced retinopathy was established, and *OPTC*-overexpressing plasmids were intravitreally injected to assess the antiangiogenesis effect of opticin. The regulatory role of opticin in integrin α2-I domain–GXXGER complex formation in vivo was analyzed by mass spectrometry. The mRNA and protein expression of RhoA/ROCK1 were examined. The concentration of Mg^2+^ as an activator of the integrin α2-I domain–GXXGER complex was measured. Solid-phase binding assays were performed to investigate the interference of opticin in integrin α2 collagen binding and the regulatory role of Mg^2+^ in that process.

**Results:**

Opticin and *OPTC*-overexpressing plasmid injection reduced retinal neovascularization in the zebrafish model of hypoxia-induced retinopathy. Mass spectrometry revealed that opticin could inhibit integrin α2-I domain–GXXGER complex formation. The Mg^2+^ concentration was also decreased by opticin, which was another indication of the complex activation. Injection of *OPTC*-overexpressing plasmids inhibited mRNA and the protein expression of RhoA/ROCK1 in the zebrafish model of hypoxia-induced retinopathy. The solid-phase binding assay revealed that opticin could block integrin α2–collagen I binding in the presence of Mg^2+^.

**Conclusions:**

Opticin exerts its antiangiogenesis effect by interfering in the Mg^2+^-modulated integrin α2-I domain–collagen complex formation and suppressing the downstream RhoA/ ROCK1 signaling pathway.

In previous studies, it was found that collagen plays a supportive role in cell proliferation and migration during neovascularization.^1^^–^^4^ In pathological retinal neovascularization, the cortical vitreous gel is invaded, and vitreous collagen is required for its growth.^5^ According to the reports from our laboratories, opticin (*OPTC*) present in vitreous exerts its antiangiogenesis effect by interfering with collagen-stimulating angiogenesis, which may be mediated by α2 and β1 integrins.^6^ However, the above findings were obtained in a previous in vitro study, and the specific interference mode and target binding site underlying the regulation of opticin remain uncertain. In this study, therefore, the zebrafish model of hypoxia-induced retinopathy was established in vivo to further investigate the specific regulatory role of opticin at the integrin-collagen binding site and its underlying mechanism.

Integrins are a large family of α and β heterodimeric receptors that mediate cell–cell and cell–extracellular matrix (ECM) interactions.^7^ It was previously confirmed that integrin α2 and β1 subunits may be the target of collagen cell binding. The integrin α2 subunit contains an additional I domain inserted into the head region, which plays a central role in collagen binding, so the potential role of the integrin α2-I domain–collagen binding complex was studied to explore the regulatory mechanism and specific interference site of opticin in this study.^7^ A specific motif recognized by the integrin α2-I domain has been identified as GXXGER in collagen, and the sequence GFOGER (where O is hydroxyproline) is the minimal recognition motif of GXXGER.^8^ Structural studies reveal that the integrin α2-I domain has two alternative conformations: open and closed. Activation of the integrin α2-I domain–GXXGER complex requires a conserved metal binding site known as the metal ion–dependent adhesion site (MIDAS) motif in the integrin α2-I domain which may be occupied by metal ions.^9^^–^^12^ The interaction of metal ions and MIDAS exposes the α7 helix at the C-terminal of the integrin α2-I domain, which opens up the collagen binding site and allows further binding to glutamic acid residues in the collagen sequence GXXGER.^13^^,^^14^ Mg^2+^ functions as a switch of the integrin α2-I domain–collagen binding complex and triggers conformational changes in the integrin α2-I domain from the closed, unliganded to the open, liganded state. As mentioned above, the interaction of Mg^2+^ and MIDAS exposes the α7 helix at the C-terminal of the integrin α2-I domain, which opens up the collagen binding site and allows further binding to glutamic acid residues in the collagen sequence GXXGER. Thus, it has been hypothesized that opticin may regulate binding of the integrin α2-I domain to collagen by interfering with Mg^2+^, which will further interfere with the collagen-stimulating angiogenesis. Because RhoA/ROCK1 is activated by integrin signals, a conduit for the transmission of signals into cells is provided.^15^ This study was conducted to investigate the role of *OPTC* in the formation of the integrin α2-I domain–GXXGER complex and the signal activities of RhoA/ROCK1.

## Experimental Procedures

### Materials and Animals

The Tg(flk:GFP) zebrafish used in this study were obtained from the Zebrafish Model Animal Facility at the Institute of Clinical and Translational Research of Sun Yat-Sen University. Reagents used for the hypoxia experiment were purchased from Sigma-Aldrich (St. Louis, MO, USA). Retinal neovascularization was visualized using a Zeiss LSM 710 confocal microscope (Carl Zeiss Meditec, Jena, Germany) and quantified using AngioTool64 (v0.6a) software (SAIC-Frederick, Frederick, MD, USA) as described before.^16^

Plasmids (pmCherry-myc-z*OPTC*-N1) for overexpression of the *OPTC* gene were used for the ocular transfection assay. The zebrafish *OPTC* gene sequence was obtained from GenBank. Plasmids were purchased from HedgehogBio (Shanghai, China).

Q Exactive Plus (Thermo Fisher Scientific, Waltham, MA, USA) was used for the mass spectrometry analysis. All materials used for mass spectrometry were obtained from Thermo Fisher Scientific. A Synergy H1 microplate reader (BioTek, Winooski, VT, USA) was used for measurement of the Mg^2+^ concentration. Antibodies used for western blotting (integrin-α2, RhoA, ROCK1, Mypt1, and phospho-Mypt1) were purchased from Bioworld Technology (Nanjing, China). Reagents used for quantitative real-time PCR were purchased from Takara Bio (Shiga, Japan). The primers were synthesized by Sangon Biotech (Guangzhou, China).

Regents used for the solid-phase binding assays were purchased from Biodragon Immunotechnologies (Beijing, China). Recombinant human opticin (NBP2-51922; Novus Biologicals, Littleton, CO, USA) and recombinant human integrin α2 (SHFS1028; Tecenet, Guangzhou, China) were used for the solid-phase binding assays. Biotinylated collagens were purchased from KYDBio Technology (Guangzhou, China).

### Zebrafish Models of Hypoxia-Induced Retinopathy

The zebrafish facility and all experimental procedures were approved by the Animal Ethical Committee of Sun Yat-sen University. The design of the hypoxia chamber and protocol for hypoxia exposure of adult zebrafish have been described previously.^16^^,^^17^ Briefly, zebrafish were first placed in normoxic water, and the O_2_ concentration was gradually reduced until final 10% air saturation over the course of 48 to 72 hours. Zebrafish were exposed to this hypoxic environment for various lengths of time (12 days at most).

### Intravitreal Injection

Before experimental operations, all zebrafish were anesthetized with 0.02% tricaine. Zebrafish were deeply anesthetized with anesthetic solution prepared in fish-tank water and placed on a moist surface under a dissecting microscope. The left eyes of the zebrafish were injected with 0.6 µL of recombinant human *OPTC* (0.5 mg/mL) or *OPTC*-overexpressing plasmids (250 mM) for 10 to 15 seconds under a dissecting microscope, and the right eyes were injected with an equivalent volume of PBS. They fully recovered from anesthesia in fish-tank water for 5 to 10 seconds, and the recovery rate was 100%.

### Retinal Preparation and Confocal Analysis

Seven days after the injection, the zebrafish were sacrificed by a lethal dose of tricaine, followed by decapitation. The retina was carefully cut four times into four equal quadrants from the edge toward the center using sharp microsurgery forceps. Retinal neovascularization was visualized under the Zeiss LSM 710 confocal microscope and quantified using AngioTool64 (v0.6a) software as described before.^16^^,^^18^

### Mass Spectrometry Analysis

All samples containing the protein were collected in each group, and their purity was validated with SDS-PAGE (180 V and 120 mA for 40 minutes) after denaturation at 75°C in the presence of lithium dodecyl sulfate buffer. Gels were stained with SimplyBlue stain for the visualization of protein bands. Strong bands corresponding to the target proteins including collagen and integrin α2 on SDS-PAGE were cut and digested by trypsin. The peptides were desalted by StageTips, vacuum dried, dissolved in 1% formic acid, and loaded on a nanoflow HPLC system coupled to the Q Exactive Plus mass spectrometer equipped with a nano-electrospray ionization source. Data-dependent acquisition was enabled, and higher energy collisional dissociation of the top five ions was performed in each cycle from the survey scan over the mass-to-charge (m/z) range of 350 to 1500 with dynamic exclusion time of 10 seconds. There were three replicates per sample. Spectra were searched with PEAKS software in the SwissProt database, with 10-ppm peptide mass tolerance and 0.02-Da fragment tolerance.

### Measurement of Mg^2+^ in Retina

Retinal tissue homogenate was centrifuged for 2 minutes at 4°C. The absorbance was measured at 510 nm using a microplate reader and was used to determine the Mg^2+^ concentration in tissues.

### Quantitative RT-PCR

Quantitative RT-PCR was performed as described previously. The primer sequences used for the relative expression of target genes including integrin α2, RhoA, ROCK1, and MYPT1 were obtained from PrimerBank.

### Western Blotting and Immunostaining

The protein expression of integrin α2, RhoA, ROCK1, MYPT1 and p-MYPT1 was detected by western blotting as described previously. Immunostaining of whole-mounted retinas and frozen sections for the detection of protein expressions of integrin α2, RhoA and p-MYPT1 was performed as described by Hu S et al.^19^

### Integrin Collagen-Binding Assay

To mimic integrin α2 collagen binding (through the formation of the integrin α2-I domain–GXXGER complex), solid-phase binding assays were performed as described by Le Goff et al.^20^ The key steps are briefly listed as follows:

#### Effects of Mg^2+^ on Binding

A 96-well plate was coated with integrin α2 (5 μg/mL) in TBS buffer for 1 hour. Mg^2+^ solution at different concentrations (0 to 2.5 mM) was added to the wells. Residual protein absorption sites were blocked with BSA for 1 hour. Biotinylated collagen I (0.5 μg/mL) was added to the wells, which were incubated for 3 hours. 3,3′,5,5′-Tetramethylbenzidine substrate was added to detect the absorbance.

#### Effects of *OPTC* on Binding

Following the above procedure, wells were coated with integrin α2 in TBS buffer containing 2-mM Mg^2+^, and biotinylated collagen I (0.5 μg/mL) was pre-incubated with *OPTC* (100–500 nM) for 2 hours.

### Statistical Analysis

The data were obtained from at least three independent assays and are expressed as mean ± SD. The results were compared between two groups by paired *t*-test and *t*-test. One-way ANOVA was used for multiple comparisons among different groups. The pairwise comparison between groups was performed using Dunnett's test and the Student–Newman–Keuls (SNK) and *q* test. The test level was α = 0.05, with *P* < 0.05 being considered significant. SPSS Statistics 25.0 (IBM, Chicago, IL, USA) was used for statistical analysis.

## Results

### Effect of Opticin on Hypoxia-Induced Retinal Neovascularization in Zebrafish

#### Establishment of Hypoxia-Induced Retinopathy Zebrafish Model

**Figure 1.**
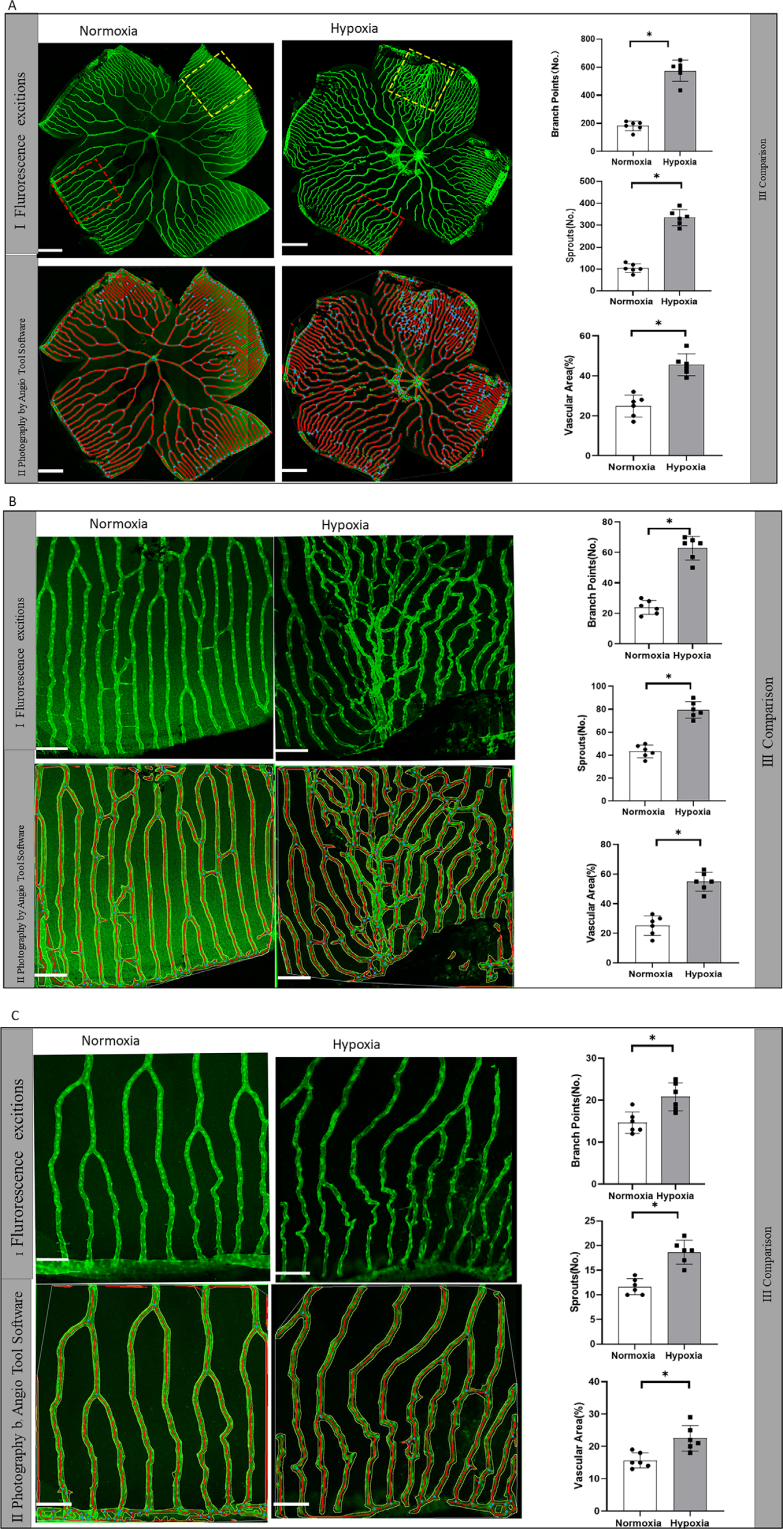
Comparison of hypoxia-induced retinal neovascularization in zebrafish. (**A**) Confocal microscopic observation of total zebrafish retinal vessels in the normoxia and hypoxia groups. (**AI**) Confocal micrograph of enhanced green fluorescent protein (EGFP)-positive vessels in zebrafish retina excited by fluorescence. The *yellow* and *red boxes* represent exemplary high and low vascularity areas of retina that are magnified and analyzed in **B** and **C**. (**AII**) Representative images of retinal vasculature of zebrafish retina analyzed by AngioTool64. The *yellow* and *red boxes* represent exemplary high and low vascularity areas of retina that are magnified and analyzed in **B** and **C**. (**AIII**) Quantification of vessel branch points, sprouts, and vascular areas in the normoxia and hypoxia groups (*P < 0.05, paired *t*-test; *n* = 6). *Scale bar*: 200 µm. (**B**): High magnification of the high vascularity area defined by the 350-µm *square yellow box* in **AI** (*P < 0.05, paired *t*-test; *n* = 6). *Scale bar*: 50 µm. (**C**) High magnification of the low vascularity area defined by the 350-µm *square yellow box* in **AI** (*P < 0.05, paired *t*-test; *n* = 6). *Scale bar*: 50 µm.

To study retinal neovascularization in a pathological setting, the zebrafish model of hypoxia-induced retinopathy was established as described by Ziquan Cao et al.^16^ The retinal capillary plexuses of arterioles and veins after hypoxia exposure for 14 days formed new sprouts that grew to a high density of capillary networks. Quantitative analysis results showed that the hypoxia group had significantly increased vascular branches, sprouts, and vascularization areas in high and low vascularity areas in retina compared with the normoxia group, suggesting that the retinal neovascularization model was successfully established (*P* < 0.05, paired *t*-test) (Figs. 1A–1C).

#### Opticin Treatment Rescued Hypoxia-Induced Angiogenesis in Zebrafish Retina

**Figure 2.**
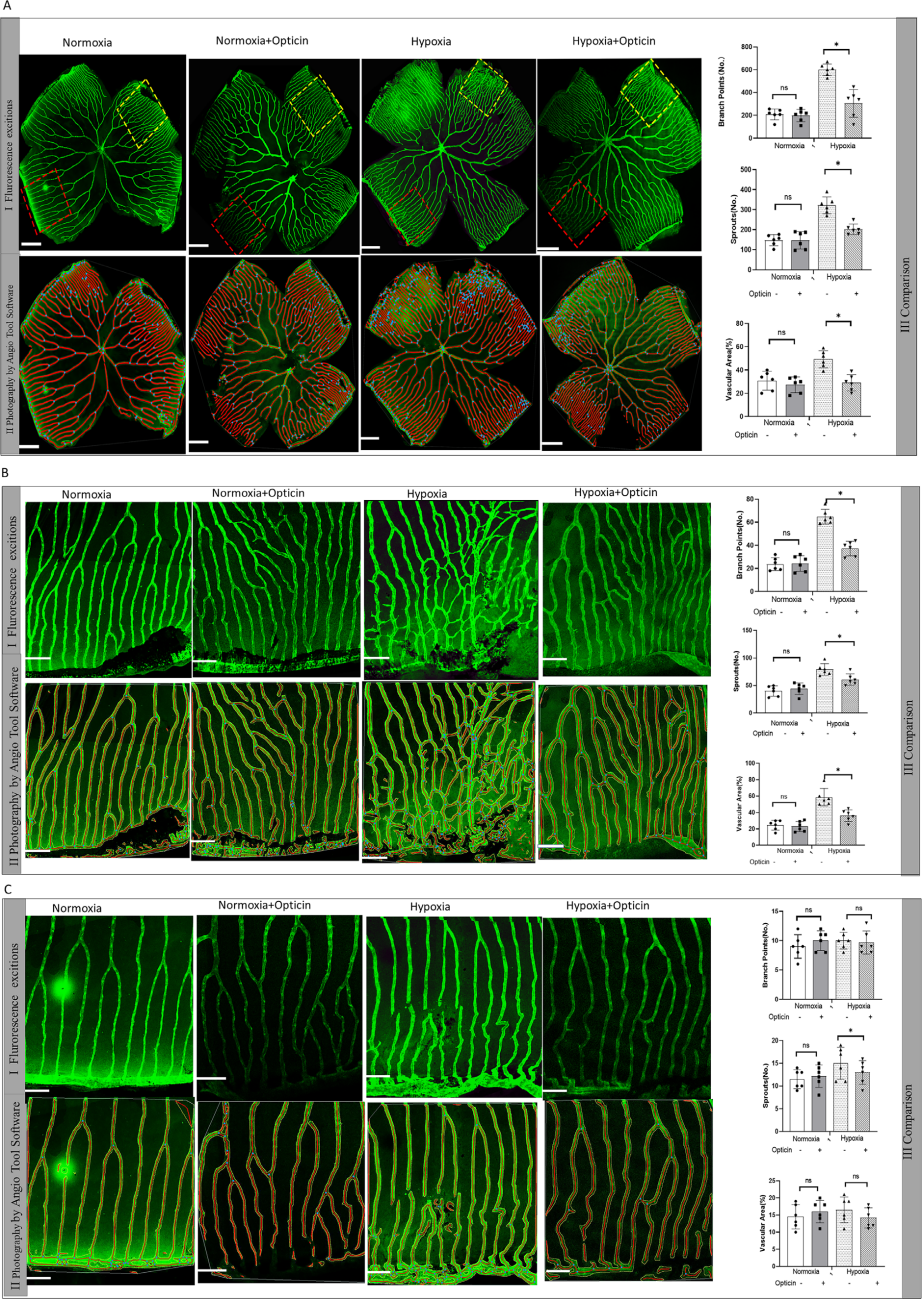
Effect of opticin on hypoxia-induced retinal neovascularization in zebrafish. (**A**) Confocal microscopic observation of total zebrafish retinal vessels in different groups. (**AI**) Confocal micrograph of EGFP-positive vessels in zebrafish retina excited by fluorescence. The *yellow* and *red boxes* represent exemplary high and low vascularity areas of retina that are magnified and analyzed in **B** and **C**. (**AII**) Representative images of retinal vasculature of zebrafish retina analyzed by AngioTool64. The *yellow* and *red boxes* represent exemplary high and low vascularity areas of retina that are magnified and analyzed in **B** and **C**. (**AIII**) Quantification of vessel branch points, sprouts, and vascular area in different groups (*P < 0.05, Dunnett's test; not significant; *n* = 6). *Scale bar*: 200 µm. (**B**) High magnification of the high vascularity area defined by the 350-µm *square yellow box* in **AI** (*P < 0.05, Dunnett's test; *n* = 6; not significant). *Scale bar*: 50 µm. (**C**) High magnification of the low vascularity area defined by the 350-µm *square yellow box* in **AI** (*P < 0.05, Dunnett's test; not significant; *n* = 6). *Scale bar*: 50 µm.

To investigate whether opticin regulates angiogenesis under pathological conditions, an intravitreal opticin injection experiment was performed. First, we assessed retinal angiogenesis under normoxia condition and found that there were no differences after opticin injection for 7 days compared with normoxia controls (Fig. 2A). Next, quantification analysis of vascular branches, sprouts, and vascularization areas of the total retina and high vascularity areas of the retina after opticin injection showed significant increase compared with the hypoxia group (Figs. 2A–2C) (*P* < 0.05, paired *t*-test; not significant).

#### *OPTC* Overexpression Reduced Hypoxia-Induced Retinal Neovascularization in Zebrafish

**Figure 3.**
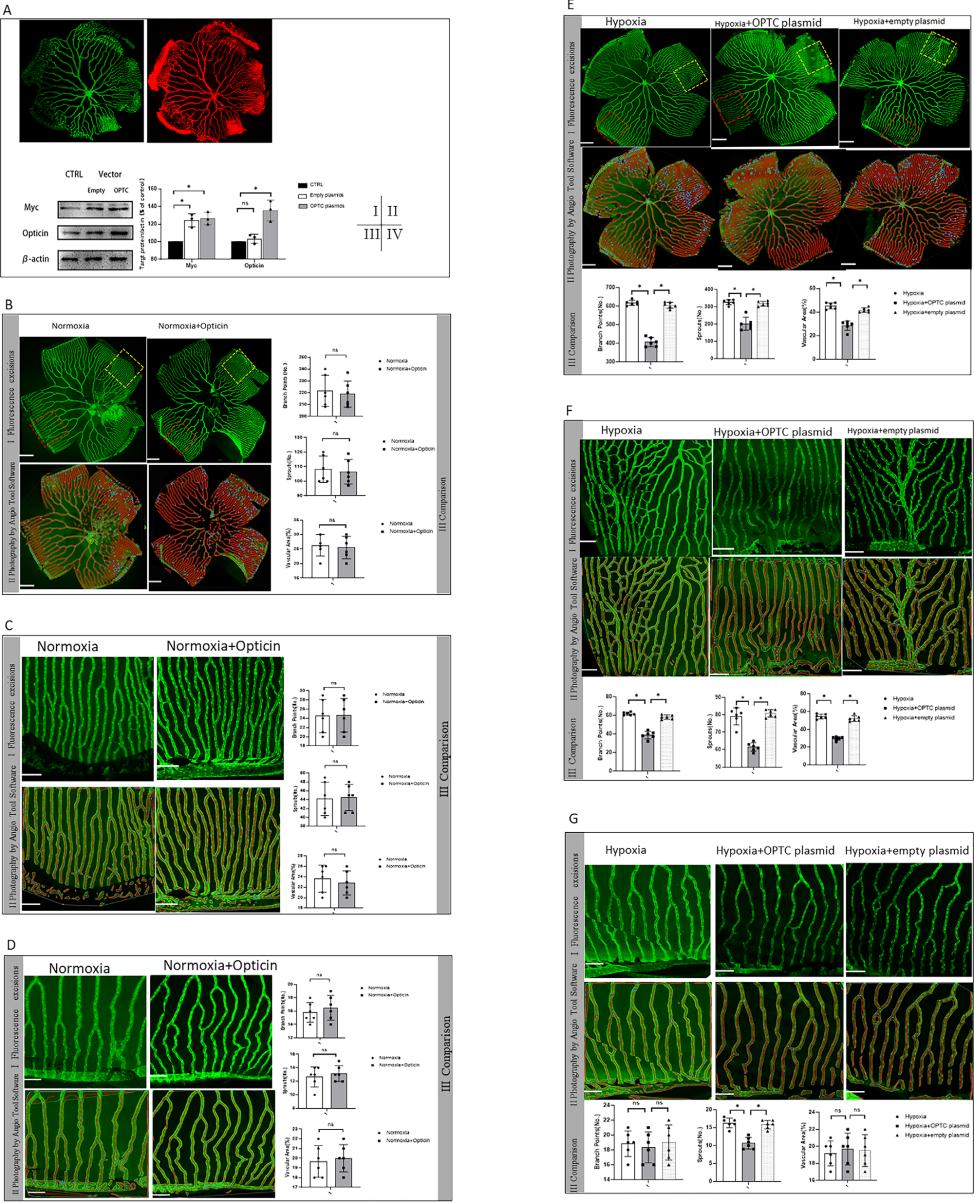
Effect of *OPTC* overexpression on hypoxia-induced retinal neovascularization in zebrafish. (**A**) Visualization of green fluorescence staining of EGFP of zebrafish retinal vessels. (**AII**) Visualization of red fluorescence by transfection of zebrafish retina. (**AIII**, **AIV**) Representative western blot image and quantification analysis of opticin and Myc protein expression changes in the retina after plasmids injection in zebrafish (*P < 0.05, Dunnett's test, not significant). (**B**) Comparison of the total zebrafish retinal vessels in the opticin injection group and normoxia controls. Quantification comparison of branch points, sprouts, and vascular area are shown on the right (*P < 0.05, paired *t*-test, not significant; *n* = 6). *Scale bar*: 200 µm. (**C**) High magnification of the high vascularity area defined by the 350-µm *square yellow box* in **BI** (*P < 0.05, paired *t*-test, not significant; *n* = 6). *Scale bar*: 50 µm. (**D**) High magnification of the low vascularity area defined by the 350-µm *square yellow box* in **BI** (*P < 0.05, paired *t*-test, not significant; *n* = 6). *Scale bar*: 50 µm. (**E**) Comparison of the total zebrafish retinal vessels in the *OPTC* overexpressing plasmids injection group, empty plasmids injection control group, and hypoxia control group. (*P < 0.05; Dunnett's test, not significant; *n* = 6). *Scale bar*: 200 µm. (**F**) High magnification of the high vascularity area defined by the 350-µm *square yellow box* in **BI** (*P < 0.05, Dunnett's test, not significant; *n* = 6). *Scale bar*: 50 µm. (**G**) High magnification of the low vascularity area defined by the 350-µm *square yellow box* in **BI** (*P < 0.05, Dunnett's test, not significant; *n* = 6). *Scale bar*: 50 µm.

To further investigate the endogenous role of opticin in regulating hypoxia-induced angiogenesis, we performed an in vivo transfection procedure utilizing *OPTC* overexpressing plasmids labeled by mCherry. Inclusion of the entire OPTC coding sequence was built in the pmCherry-myc-z*OPTC*-N1 plasmid (Supplementary Fig. S1A). Empty plasmids (pmCherry-myc-C1) were built as control. First, we used the 293T cell line to measure the transfection efficiency of plasmids. Results showed that both *OPTC* overexpressing plasmids and empty plasmids were successfully constructed with red fluorescence-positive staining of 293T cells (Supplementary Figs. S1B, S1C). Then, after plasmid injection, the red fluorescence of both *OPTC* overexpressing plasmids and empty plasmids (Supplementary Figs. S1D, S1E) was visualized when we focused the vessel layer of the whole-mount retina slide (Figs. 3AI, 3AII). In addition, the results of western blotting showed that the plasmids could produce *OPTC* and Myc proteins, further validating the successful construction of plasmids (Figs. 3AIII, 3AIV) (*P* < 0.05, Dunnett's test).

It was found that the *OPTC* overexpression group had significantly decreased vascularization areas, branches, and sprouts in retina compared with the hypoxia group and the empty plasmid injection group (Fig. 3B) (*P* < 0.05, Dunnett's test). Similarly, subanalysis of different vascularity areas showed that the branches, sprouts, and vascularization areas in the *OPTC* overexpression group were significantly decreased in high vascularity areas, whereas only sprouts and vascularization areas were significantly decreased in low vascularity areas compared with the hypoxia group and the empty plasmid injection group (Figs. 3C, 3D) (*P* < 0.05, Dunnett's test). These results suggest that overexpression of *OPTC* could suppress retinal neovascularization in zebrafish.

### Effect of Opticin on Formation of the Integrin α2-I Domain–Collagen Complex

#### Opticin Downregulated Integrin α2-I Domain and GXXGER Expression in Zebrafish Retina

**Figure 4.**
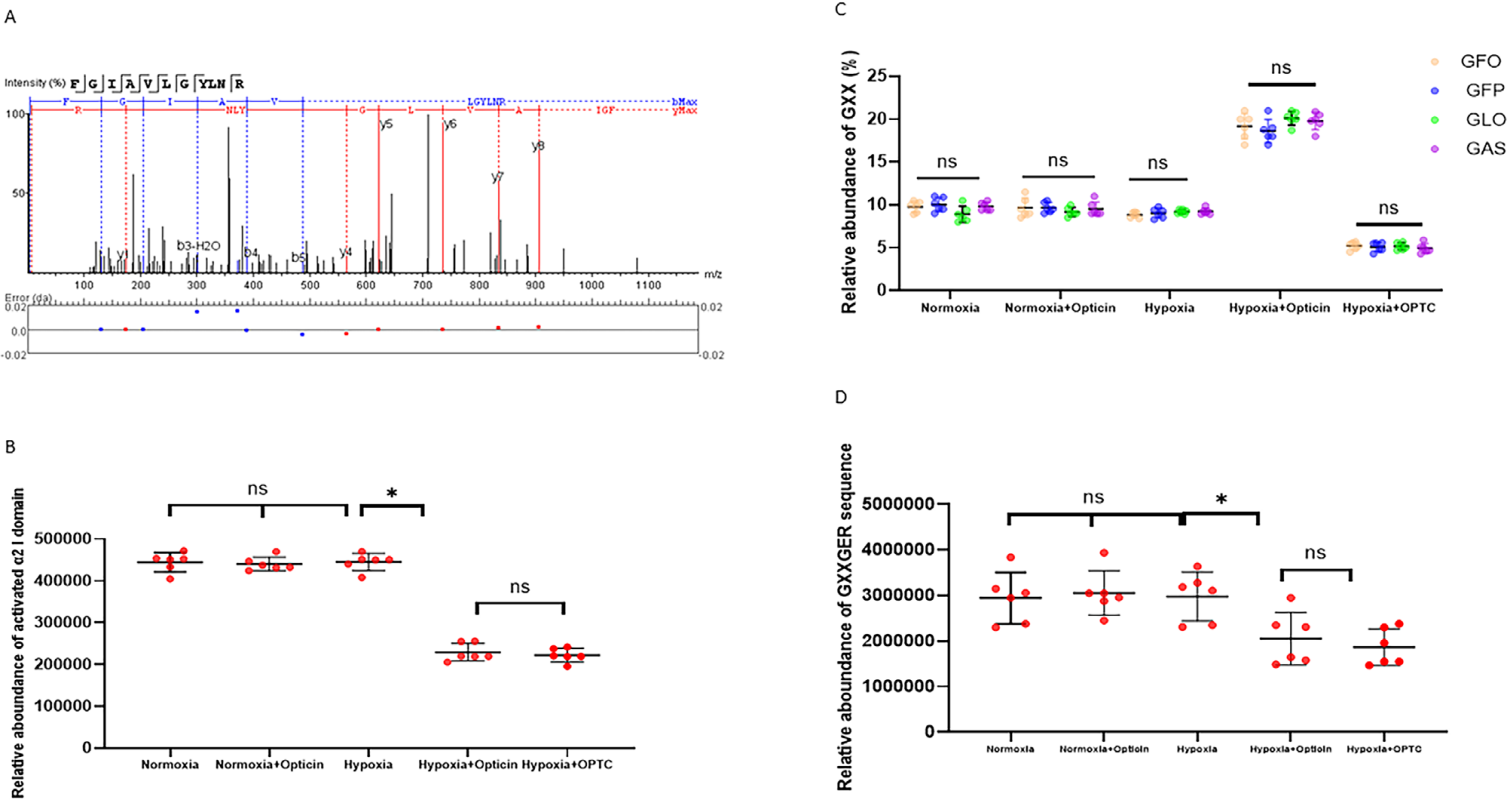
Effect of opticin on integrin α2-I domain-GXXGER complex formation in zebrafish. (**A**) Representative image of summed tandem mass spectrometry spectra of integrin α2-I domain peptide covering the GYLNR motif. (**B**) Quantitative analysis of the abundance of the GYLNR peptide in different groups. the data point represents one liquid Chromatography with tandem mass spectrometry (LC-MS/MS) run (*P < 0.05, Dunnett's test; not significant). (**C**) Quantitative analysis of the abundance of the GXX sequence calculated by dividing the number of unique tandem mass spectra matching to a peptide containing GXX sequences to the peptides containing GXXGER sequences in different groups. The data point represents one LC-MS/MS run. (**D**) Quantitative analysis of the abundance of the GXXGER peptide in different groups. The data points represent one LC-MS/MS run (*P < 0.05, Dunnett's test, not significant).

To investigate the regulatory role of opticin in the formation of the integrin α2-I domain–collagen complex in vivo, the expression of activated forms of the integrin α2-I domain and GXXGER peptides was measured using mass spectrometry to monitor the expression of amino acid sequences. Comparison of the closed and open forms of integrin α2-I domain structures showed that the αC helix provided a steric barrier in the closed conformation for attainment of the metal–ligand bond. The open conformation was seen in the presence of ligands, whereas the closed conformation was seen in the absence of ligands. Residues corresponding to the ligand were Gly284–Arg288 (GYLNR). Therefore, the peptide FGIAVLGYLNR covering the GYLNR motif was identified as the open form of the integrin α2-I domain in the samples. The best unique amino acid sequences of GYLNR were confirmed by matrix-assisted laser desorption/ionization–time of flight (MALDI-TOF) in the samples (Fig. 4A). Comparisons of the relative abundance of this sequence showed that there was no statistical significance between the normoxia and hypoxia groups, indicating that hypoxia did not induce activation of the integrin α2-I domain (*P* > 0.05, SNK *q*-test). Also, our results showed that opticin had no effect on activating integrin α2-I domain expression under the normoxia condition (*P* > 0.05, SNK *q*-test). There were significant differences in samples in the comparisons among the hypoxia, opticin injection, and *OPTC* overexpression groups (Fig. 4B) (*P* < 0.05, SNK *q*-test). The above findings suggest that activation of the integrin α2-I domain was reduced by opticin. Also, the best unique amino acid sequences of GXXGER on collagen were confirmed by MALDI-TOF, and relative abundance values were detected (Figs. 4C–4F). The mass spectrometry analysis revealed that there were indistinguishable differences among the peptides containing the first triplet of individual hydroxylated integrin α2-I domain binding motifs (GFO, GFP, GLO, GAS) in samples in different groups (Fig. 4G) (*P* > 0.05, one-way ANOVA). The content of GXXGER sequences in samples was significantly decreased with increasing concentrations of opticin (Fig. 4H) (*P* < 0.05, SNK *q*-test).

#### Opticin Inhibited Integrin α2 Collagen Binding in the Solid-Phase Binding Assay

**Figure 5.**
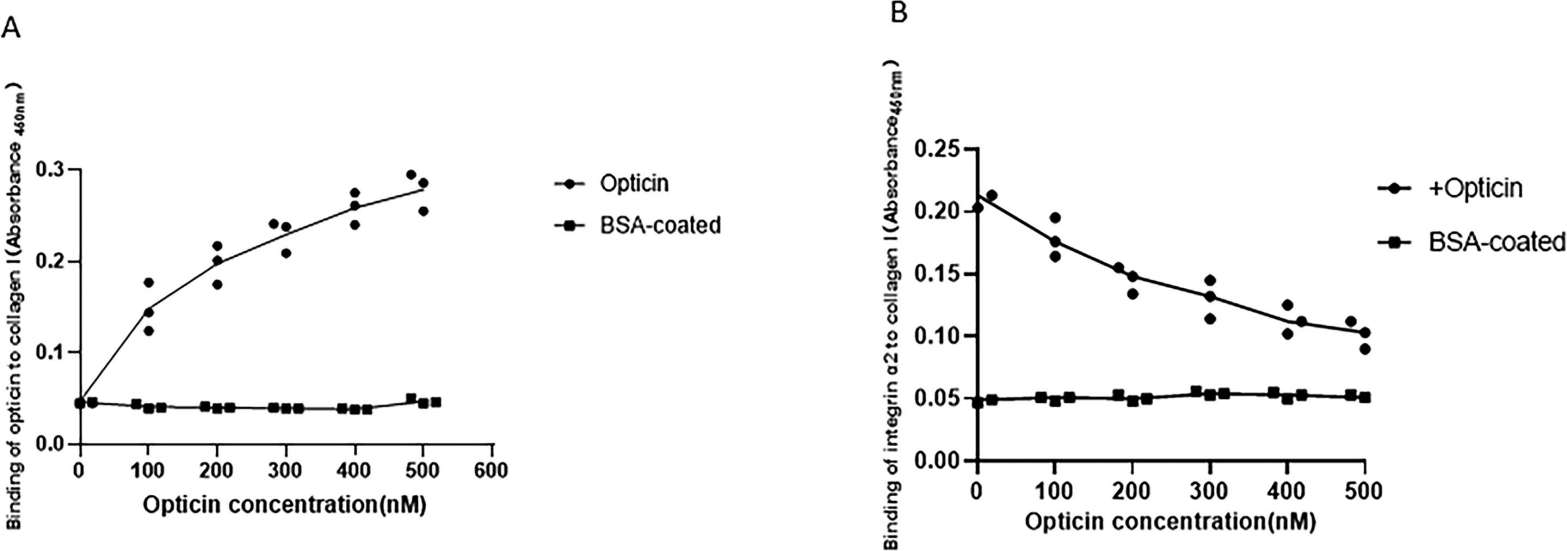
Effect of opticin on integrin α2–collagen I binding in solid-binding assays. (**A**) Opticin-Collagen I solid-binding assays. Solid-state analysis of recombinant opticin binding to recombinant collagen I immobilized on 96-well plates. HRP-conjugated streptravidin reflected by absorbance was used to detect the bound opticin, and BSA was used as a background value. The binding of opticin to collagen I increased gradually as the concentration increased. All experiments were in triplicate. Each point represents the mean of three determinations. (**B**) Integrin α2 -collagen I inhibition study by opticin. Solid-state analysis of recombinant integrin α2 binding to recombinant collagens that incubated with recombinant opticin on 96-well plates. HRP-conjugated streptravidin reflected by absorbance was used to detect the bound opticin, and BSA was used as a background value. With increasing opticin concentration, binding of integrin α2 to collagen I decreased gradually. All experiments were in triplicate. Each point represents the mean of three determinations.

To directly explore the inhibition of integrin α2 collagen binding by opticin (through the formation of integrin α2-I domain–GXXGER complexes), solid-phase assays were performed using purified recombinant proteins. Collagen I was biotinylated as a label. Ligands including integrin α2 and opticin binding to biotinylated collagen I were detected based on the absorbance detected using horseradish peroxidase (HRP)-conjugated streptavidin. The absorbance changes were characterized as a marker for the binding affinity of proteins. Inhibition experiments were performed using a fixed concentration of collagen I and a fixed concentration of Mg^2+^. BSA-coated wells were identified as blank controls. The results revealed that opticin could specifically bind to collagen I in a concentration-dependent manner (Fig. 5A). Inhibition studies were performed. Collagen I was incubated with recombinant opticin on 96-well plates, and solid-state analysis detected integrin α2 binding to recombinant collagen I. HRP-conjugated streptravidin reflected by absorbance was used to detect the bound integrin α2, and BSA was used as a background value. Results indicate that, with an increase in opticin concentration, the binding of integrin α2 to collagen I decreased gradually, suggesting that opticin inhibited integrin α2 collagen I based on the increasing concentration of opticin. It can be seen that opticin inhibited integrin α2–collagen I binding in a dose-dependent manner at a fixed concentration of Mg^2+^ (Fig. 5B).

#### Role of Mg^2+^ in the Formation of Integrin α2-I Domain–Collagen Complex and Opticin Regulation

**Figure 6.**
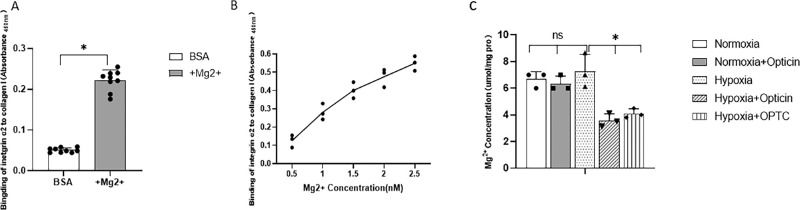
The role of Mg^2+^ in integrin α2–collagen complex formation and opticin regulation. (**A**) The role of Mg^2+^ in the integrin α2 -collagen I solid-binding assay. Solid-state analysis of recombinant integrin α2 binding to recombinant collagen I immobilized on 96-well plates under different conditions. HRP-conjugated streptravidin reflected by absorbance was used to detect the bound opticin, and BSA was used as a background value. The BSA control without Mg^2+^ showed rare binding, whereas the binding of integrin α2 to collagen I increased significantly in the presence of Mg^2+^, which means that integrin α2–collagen I binding was significantly facilitated by the presence of Mg^2+^ (*P* < 0.05, *t*-test). Each sample was measured in three parallel wells. All experiments were in triplicate. Each point is an individual data point. (**B**) Concentration-dependent effect of Mg^2+^ on integrin α2–collagen I binding. Solid-state analysis of recombinant integrin α2 binding to recombinant collagen I immobilized on 96-well plates at different concentrations of Mg^2+^. HRP-conjugated streptravidin reflected by absorbance was used to detect the bound integrin α2. As the Mg^2+^ concentration increased, the binding of integrin α2 to collagen I increased gradually. All experiments were in triplicate. Each point represents the mean of three determinations. (**C**) Quantitative analysis of Mg^2+^ concentration in different groups (*P* < 0.05, SNK *q*-test, not significant). All experiments were in triplicate. Each point is an individual data point.

To further define the role of Mg^2+^ in integrin α2 collagen binding, solid-binding assays were performed, and in vivo formation of integrin α2 collagen complexes was mimicked using purified recombinant proteins. Collagen I at a fixed concentration was added to integrin α2-coated wells. The results of solid-binding assays showed that there was significantly more integrin α2–collagen I binding in the presence of Mg^2+^ compared with the BSA-coated group (Fig. 6A) (*P* < 0.05, *t*-test). The results indicate that integrin α2 bound to collagen I with high affinity in the presence of Mg^2+^.

Next, the ability of integrin α2 to bind to collagen I was examined in the presence of Mg^2+^ at various concentrations. It was found that integrin α2 bound to collagen I in a dose-dependent manner (Fig. 6B), indicating that integrin α2–collagen I binding was facilitated by Mg^2+^.

Considering the paramount importance of Mg^2+^ in integrin α2–collagen binding, whether opticin regulates the formation of integrin α2-collagen complex via regulation of Mg^2+^ was investigated. To further clarify the in vivo function of Mg^2+^, Mg^2+^ concentrations in zebrafish retina were analyzed, and the results indicate that Mg^2+^ was decreased by opticin and the injection of *OPTC* overexpressing plasmids (Fig. 6C) (*P* < 0.05, SNK *q*-test). The findings demonstrate that opticin negatively regulates Mg^2+^ during pathological angiogenesis in vivo.

### Effect of Opticin on RhoA/ROCK1 Signaling Pathway

Given that the RhoA/ROCK1 signaling pathway plays a critical role in regulating angiogenesis activated by integrins, and that it was previously demonstrated in vitro that opticin can affect the bioactivity of human retinal vascular endothelial cells (hRVECs), which may be regulated by the RhoA/ROCK1 signaling pathway, we explored the status of the RhoA/ROCK1 signaling pathway in vivo in response to opticin/*OPTC* overexpressing plasmid injection.

**Figure 7. fig7:**
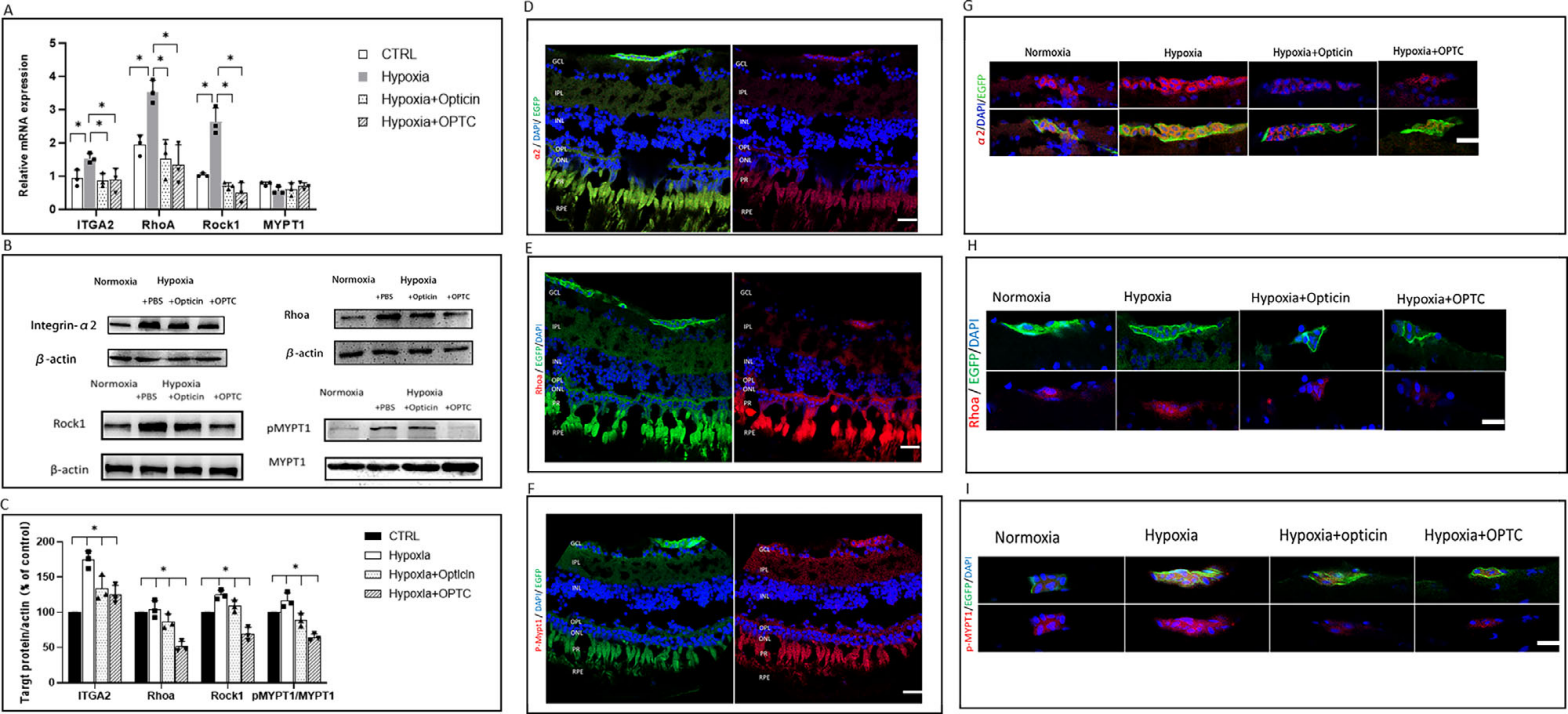
Effect of opticin on RhoA/ROCK1 signaling in zebrafish retina. (**A**) Opticin reduced the hypoxia-induced increase of integrin α2, RhoA, and ROCK1 mRNA expression in zebrafish retina; 28S rRNA was used as housekeeping gene (*P < 0.05, SNK *q*-test, not significant). (**B**) Representative protein bands and corresponding quantitative bar plots of integrin α2, RhoA, ROCK1, and p-MYPT1. β-Actin confirmed equal loading. (**C**) Corresponding quantitative bar plots of integrin α2, RhoA, ROCK1, and p-MYPT1. β-Actin confirmed equal loading. Opticin reduced the hypoxia-induced increase in integrin α2, RhoA, ROCK1, and p-MYPT1 protein expression in zebrafish retina. β-Actin confirmed equal loading (*P < 0.05, SNK *q*-test, not significant). (**D**) Visualization of integrin α2 (*red*) in the retinal section. *Scale bar*: 20 µm. (**E**) Visualization of RhoA (*red*) in the retinal section. *Scale bar*: 20 µm. (F) Visualization of p-MYPT1 (*red*) in the retinal section. *Scale bar*: 20 µm. (**G**) Magnified visualization of integrin α2 (*red*) in the retinal cell. *Scale bar*: 10 µm. (**H**) Magnified visualization of RhoA (*red*) in the retinal cell. *Scale bar*: 10 µm. (**I**) Magnified visualization of p-MYPT1 (*red*) in the retinal cell. *Scale bar*: 10 µm. Opticin/OPTC downregulated hypoxia-induced upregulation of integrin α2, RhoA, and p-MYPT1 expression in the endothelial cells of zebrafish retina. GCL, ganglion cell layer; IPL, inner plexiform layer; INL, inner nuclear layer; OPL, outer plexiform layer; ONL, outer nuclear layer. *Blue* (DAPI) indicates cell nuclei.

To clarify the regulatory role of opticin in the RhoA/ROCK1 signaling pathway following the formation of the integrin α2-I domain–collagen complex, zebrafish retinas were isolated, and the expression of target genes was detected at both transcriptional and protein levels. As shown in [Fig fig7]A, hypoxia promoted the mRNA expression of *RhoA* and *ROCK1*, two downstream target genes of the RhoA/ROCK1 signaling pathway, as well as integrin α2. On the other hand, the expressions of these genes was downregulated by opticin injection and *OPTC* overexpression (*P* < 0.05, SNK *q*-test). The mRNA expression of MYPT1 was not changed due to the phosphorylation of MYPT1 at the protein level (*P* > 0.05, SNK *q*-test). These results suggest that opticin negatively regulated activation of the RhoA/ROCK1 signaling pathway following formation of the integrin α2-I domain–collagen complex in zebrafish retina. Similarly, the protein expression of the integrin α2 and RhoA/ROCK1 signaling pathway showed the same trend. Hypoxia induced significantly increased expression of integrin α2, RhoA, and ROCK1 at the protein level. The expression of p-MYPT1, a marker for ROCK1 activation in the intracellular domain, was also downregulated by opticin at the protein level (Figs. 7B–7C) (*P* < 0.05, SNK *q*-test).

To confirm that the above-mentioned signal changes in zebrafish retina, immunofluorescence staining was performed in cells. The expression levels of target proteins in cells of retinal sections were detected in each group using immunofluorescence staining (Supplementary Fig. S2). Specific protein staining was observed in cells costained by enhanced green fluorescent protein (EGFP) in retinal sections, and the staining intensities of integrin α2, RhoA, and p-MYPT1 proteins in the hypoxia group were significantly higher than in the normoxia group, suggesting an enhanced RhoA/ROCK1 signaling pathway in the hypoxia group. Opticin or *OPTC* overexpressing plasmid injection significantly inhibited the protein expressions of integrin α2, RhoA, and p-MYPT1 (Figs. 7D–7I). To sum up, opticin negatively regulated the RhoA/ROCK1 signaling pathway following the formation of the integrin α2-I domain–collagen complex in neovascularization in zebrafish.

## Discussion

It is widely accepted that the vitreous body generally resists angiogenesis, and many of the known ECM molecules play central roles in maintaining the angiogenesis balance in the vitreous body.^5^ Previously, it was confirmed that collagen, a major ECM component of vitreous gel, provides a supporting scaffold for angiogenesis in preretinal neovascularization diseases such as proliferative diabetic retinopathy.^1^^,^^4^^,^^5^ Opticin, a kind of ECM glycoprotein, is thought to colocalize with the collagen fibers and probably has an inhibitory effect on angiogenesis. It has been recently found that *OPTC* can inhibit collagen-induced promotion of bioactivities of hRVECs.^6^ To further study the regulation role of opticin on collagen-induced neovascularization in vivo and its potential mechanism, we generated a hypoxia-induced retinopathy zebrafish model. In comparison with the oxygen-induced retinopathy mouse model, the availability of vasculature-specific transgenic lines of zebrafish model allowed more convenient observation of retinal vessels in a disease context,^16^^,^^21^ such as intended in this study. Moreover, the retinal vasculature of zebrafish constitutes a membranous layer directly attached to the vitreous interface and covering the inner limiting membrane, which makes it more appropriate for mimicking preretinal neovascularization disease.^21^ In this study, the results showed that hypoxia/ischemia–mediated retinal damage reached a peak at 7 days after hypoxia in the zebrafish model, consistent with the research results by Ziquan Cao et al.^16^ Significantly greater preretinal neovascularization was observed in the hypoxia models compared with the normoxia groups. These observations suggest that this model could provide a unique opportunity to study kinetically the development of preretinal neovascularization in adult animals and to assess the therapeutic efficacy of active antiangiogenic drugs.

Next, our results suggest that opticin could inhibit hypoxia-induced retinal neovascularization. Moreover, the reduction in pathological angiogenesis due to opticin is further supported by the observations of impaired angiogenic response to *OPTC* gene overexpression from the endogenous level. Our study is in line with previous studies reporting that *OPTC*-deletion mice exhibited increased preretinal neovascularization in oxygen-induced retinopathy models.^22^ Specifically, data from our ocular transfection experiment provide evidence that the protective effect of opticin against retinal angiogenesis could be achieved by gene overexpression in vivo. These results have major clinical and therapeutic significance, as preretinal neovascularization disease is a leading cause of vision impairment and blindness in human ophthalmic diseases. Current treatments for these diseases, including anti-vascular endothelial growth factor therapy and the standard care for retinal neovascularization, are invasive, and many patients do not respond very well. This study suggests that opticin may be a promising therapeutic target for preretinal neovascularization disease at both exogenous and endogenous levels.

Some previous work has attempted to illustrate how opticin inhibits interactions between collagen and integrin in Matrigel, mimicking the collagen environment, but there have been no direct investigations of the underlying mechanism of the antiangiogenic activity of opticin in actual animal models.^20^ Our observations provide the first experimental evidence, to the best of our knowledge, that opticin can specifically interfere with conformation of the integrin α2-I domain–GXXGER complex in vivo. The primary evidence supporting this conclusion includes the following: (1) Based on mass spectrometry analysis, the amino acid expression of activated forms of the integrin α2-I domain covering the Gly284–Arg288 (GYLNR) sequence and its binding motifs in collagen identified as the GXXGER sequence are both reduced by opticin in zebrafish retina. (2) Based on our in vivo study results, opticin specifically inhibits α2 integrin–collagen binding affinity in solid-binding assays. Because it has been well documented that the integrin-collagen binding site is the integrin α2-I domain and GXXGER in collagen, the solid-binding assays results are another indication of integrin α2-I domain–GXXGER complex formation. (3) The concentration of Mg^2+^ in zebrafish retina was downregulated by opticin treatment. On the one hand, it was further confirmed in a previous in vitro study that opticin inhibits collagen-induced promotion of bioactivities of hRVECs via integrins. On the other hand, a new specific interference mode and target binding site were found to underlie the antiangiogenic mechanism of opticin, extending previous research by Le Goff et al.^23^

It has been previously reported that integrin α2–collagen binding through MIDAS is dependent on Mg^2+^ levels in the ECM.^5^ Our current study shows that the concentration of Mg^2+^ was downregulated by opticin treatment, thus adding another dimension to our understanding of the intricate activation of the integrin α2-I domain–collagen complex. There is a possibility that opticin decreases the concentration of Mg^2+^ and then downregulates activation of the integrin α2-I domain–collagen complex. It also seems reasonable to speculate that opticin–collagen binding could trigger large conformational changes in the complex, thus making the MIDAS more inaccessible for the Mg^2+^ and ligand. Neither of the two hypotheses can be excluded, but they both stress the importance of the formation of integrin α2-I domain–collagen complex. These possibilities were further verified by the solid-binding results indicating that integrin α2–collagen binding affinity was facilitated by Mg^2+^ in a dose-dependent manner. It was also demonstrated that opticin specifically inhibited integrin α2–collagen binding affinity, consistent with the in vivo study results. It is worth noting that solid-binding assays can be used to investigate the activation state of the complex in the presence of Mg^2+^, which may add another dimension to research on the role of the integrin α2–collagen binding complex. Thus, this study warrants further investigation of the relationship between Mg^2+^ concentration and opticin and further conformational changes of the complex in pathological conditions.

The results of this study demonstrate that formation of the integrin α2–collagen complex is indispensable for the antiangiogenesis effect of opticin, and regulation of the RhoA/ROCK1 signaling pathway also played an essential role. Although the significance of the RhoA/ROCK1 signaling pathway has been well recognized in preretinal neovascularization diseases,^15^ its relevant regulation in hypoxia-induced retinopathy has not been clearly explored. Our previous in vitro study demonstrated that opticin could affect the bioactivity of hRVECs, which may be regulated by integrins and RhoA/ROCK1 signaling. The correlation between the integrin α2 and RhoA/ROCK1 signaling pathways has not been prudently explored. Evidence obtained in our current study from mRNA and the protein expression of both integrin α2 and other target molecules of RhoA/ROCK1 signaling in retina supports the conclusion that RhoA/ROCK1 signaling is a common downstream effector responsible for mediating the cellular responses of integrin α2 on pathological angiogenesis. Our study is in line with our previous in vitro study, which found that opticin is an inhibitor of RhoA/ROCK1 signaling in hypoxia-induced angiogenesis regulation. To sum up, this study suggests that opticin downregulates activation of the integrin α2-I domain–collagen complex, leading to such intracellular responses as increased RhoA/ROCK1 activity and actin stress fiber formation.

In summary, the research results provide compelling evidence that opticin is an important regulator of pathological angiogenesis by modulating the formation of the integrin α2-I domain–collagen complex and further suppressing the RhoA/ROCK1 signaling pathway. These results, coupled with the finding that pharmacological overexpression of the *OPTC* gene prevents hypoxia-induced neovascularization, suggest that opticin-specific inhibitors can be developed as promising therapeutic targets for the treatment of diseases associated with abnormal angiogenesis such as retinal vascular disorders and other proliferative vascular diseases.

## Supplementary Material

Supplement 1
