# Supplementary material for: Opticin Ameliorates Hypoxia-Induced Retinal Angiogenesis by Suppression of Integrin α2-I Domain–Collagen Complex Formation and RhoA/ROCK1 Signaling
Source: Invest Ophthalmol Vis Sci. 2022 Jan 10;63(1):13. doi: 10.1167/iovs.63.1.13 (PMC8762695; doi:10.1167/iovs.63.1.13)
Supplement: Supplement 1 [file iovs-63-1-13_s001.pdf]

SUPPLEMENTARY DATA

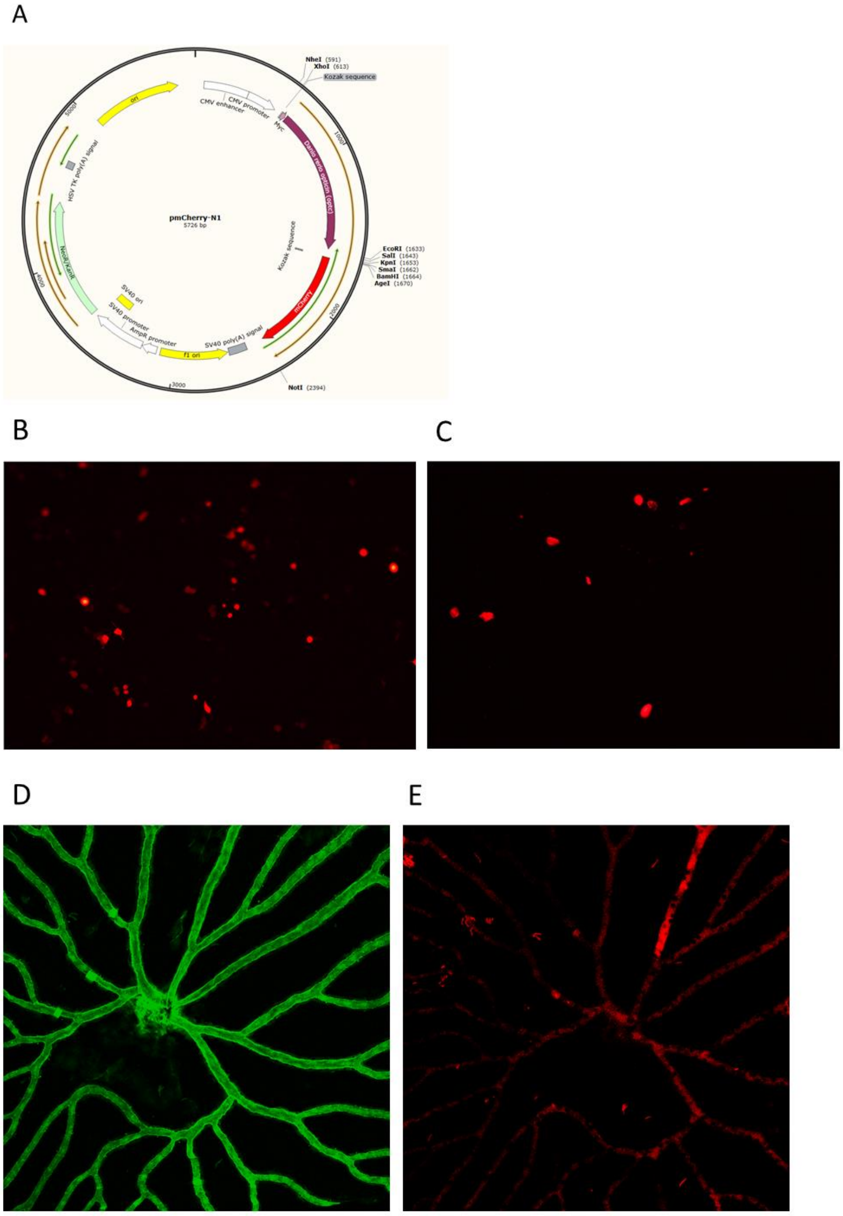

Supplementary data1 A: Successful construction of plasmids(pmCherry-myc-zOPTC-N1). B: Visualization of red label of empty plasmids plasmids by transfection of 293T cells. C: Visualization of red label of OPTC overexpressing plasmids. by transfection of 293T cells. D: Visualization of green fluorescence staining of EGFP of zebrafish retinal vessels; E: Visualization of red fluorescence by transfection of zebrafish retina.

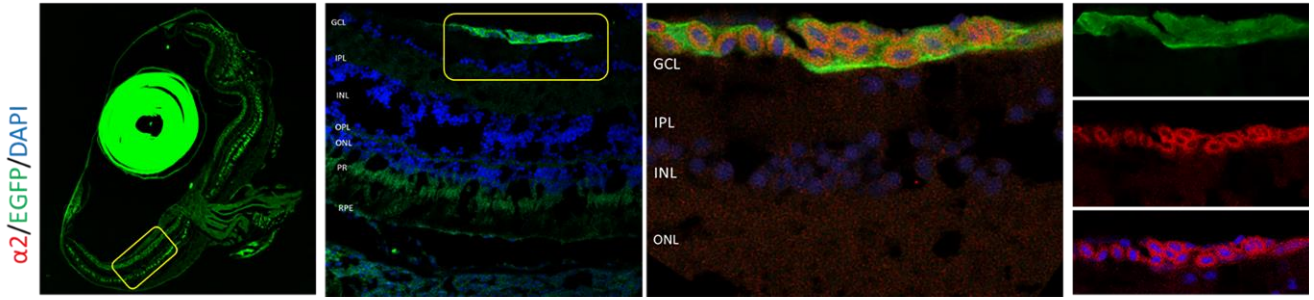

Supplementary data2 : Representative confocal scan of zebrafish retinal section. Frozen section of the whole eye of zebrafish and visualization of ECs (yellow box) in the retinal section are shown on the right. Visualization of integrin α2 (red) in the retinal section. DAPI (blue) indicates retinal layers.

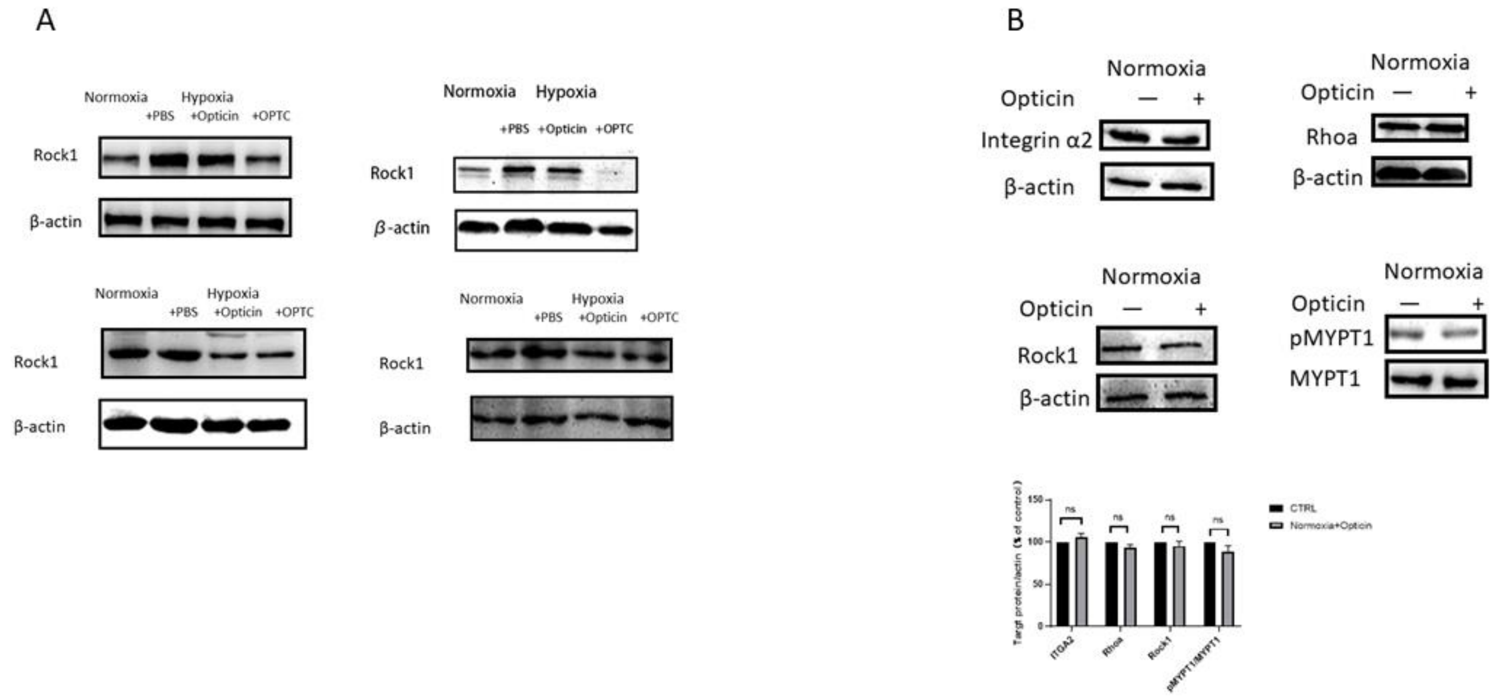

Supplementary data3 : A: Representative replicates of ROCK1 western blotting analysis .β-actin confirms equal loading. B: Representative images of western blotting analysis of integrin α2, RhoA, ROCK1 and p-MYPT1 protein expression in normoxia group and normoxia+opticin groups. β-actin confirms equal loading.
